# Blocking TNFR1 internalization and cell death represents a critical mechanism for alleviating enteritis in adult grass carp: The potential role of dietary myo-inositol in enhancing intestinal health

**DOI:** 10.1016/j.aninu.2025.12.020

**Published:** 2026-06-02

**Authors:** Meiqi Wang, Lin Feng, Pei Wu, Yang Liu, Hongmei Ren, Xiaowan Jin, Xiaoqiu Zhou, Weidan Jiang

**Affiliations:** a Animal Nutrition Institute, Sichuan Agricultural University, Chengdu 611130, Sichuan, China; b Fish Nutrition and Safety Production University Key Laboratory of Sichuan Province, Sichuan Agricultural University, Chengdu 611130, Sichuan, China; c Key Laboratory of Animal Disease-Resistance Nutrition, Ministry of Education, Ministry of Agriculture and Rural Affairs, Key Laboratory of Sichuan Province, Chengdu 611130, Sichuan, China

**Keywords:** Myo-inositol, Intestinal inflammation, Cell death, Apoptosis, Endosome, Grass carp

## Abstract

In high-density intensive aquaculture systems, enteritis has emerged as a critical health concern. In bony fish, the intestine serves as the core organ of the digestive system and plays an important part in maintaining physiological homeostasis. Disruption of intestinal barrier integrity can lead to metabolic disturbances, inflammatory responses, and progressive impairment of intestinal barrier function. Myo-inositol (MI), a vitamin-like nutrient, exerts notable beneficial effects on the structural integrity of physical barriers and non-specific immunity in various aquatic species. A total of 450 adult grass carp (*Ctenopharyngodon idella*) (704.84 ± 0.91 g) were randomly divided into 6 treatments (3 replicates per treatment) to receive six levels of dietary MI (35 [basal diet], 98, 195, 292, 389, and 487 mg/kg). Following an 8-week feeding period, intestinal inflammation was induced using dextran sulfate sodium (DSS). Compared with the MI supplement group, the results demonstrated that MI deficiency (basal diet) suppressed intestinal development in adult grass carp, aggravated intestinal structural damage in DSS-induced enteritis, and reduced the number of intestinal goblet cells (*P* < 0.001). Moreover, MI deficiency increased the incidence of DSS-induced enteritis (*P* < 0.001). Mechanistically, MI deficiency caused sustained NF-κB p65 activation by disrupting early endosome formation and impairing TNFR1 internalization (*P* = 0.015). This cascade ultimately triggered cell apoptosis and exacerbated intestinal inflammation. Collectively, these findings revealed how MI contributes to the maintenance of intestinal barrier function and enhanced understanding of its role in regulating intestinal health in grass carp.

## 1 Introduction

In recent years, the global aquaculture industry has demonstrated a rapid growth trend. Nevertheless, enteritis has emerged as a particularly significant issue in high-density intensive aquaculture systems, with reported incidence rates ranging from 10% to 40% (Chen et al., 2020; Meng et al., 2022). In bony fish, the intestine plays an important role in maintaining physiological homeostasis. Impairment of the intestinal barrier integrity can lead to metabolic disorders, inflammatory responses, and progressive deterioration of intestinal barrier function (Turner, 2009). Consequently, the health status of the intestine is not only crucial for individual fish survival but also for the productivity and sustainability of aquaculture practices. Myo-inositol (MI), a vitamin-like nutrient, has demonstrated a significant positive impact on the physical barrier integrity and non-specific immune function in various aquatic species (Li et al., 2017; Wang et al., 2020). Findings from earlier investigations indicate that MI deficiency can impair the growth performance and immune barrier function in the intestines of both juvenile and adult grass carp (*Ctenopharyngodon idella*) (Li et al., 2018; Wang et al., 2025a, b). In addition, dietary MI deficiency exacerbates the hypertrophy and hemorrhage in the fish intestine following *Aeromonas hydrophila* infection, while also impairing the intestinal physical barrier function. These findings indicate that MI is vital for enhancing the immune function of aquatic animals. Consequently, building upon the previous research, further refinement and systematic investigation were conducted on the specific mechanisms by which MI alleviates fish enteritis.

As a pleiotropic pro-inflammatory cytokine, TNF plays a critical role in maintaining intestinal integrity; however, it also serves as a major driver of intestinal epithelial cell death (Ruder et al., 2019). Upon TNF stimulation, RIPK1 and TRADD form complex I on the plasma membrane, which mediates NF-κB signaling (Wu et al., 2024). Clathrin-mediated endocytosis of TNFR1 complex I results in the separation of complex I and subsequent creation of complex II (consisting of TRADD, deubiquitinated RIPK1, and Caspase-8), thereby inducing apoptosis (Huyghe et al., 2023). When Caspase-8 activity is inhibited, MLKL is activated, which oligomerizes and translocates to the plasma membrane to form pores (Moquin and Chan, 2010). This ultimately results in cell membrane lysis and induces necroptosis. Furthermore, endocytosis and endosomal transport serve an essential function in both the initiation and termination of TNFR1-mediated cell death signal transduction. However, their importance in modulating immune responses and inflammatory processes remains underappreciated. Therefore, it is essential to explore the role of MI in modulating TNFR1 internalization within the context of intestinal inflammation.

The EFHD2 is a calcium-binding adaptor protein initially discovered in lymphocytes. It is a multifunctional protein molecule predominantly expressed in the immune system and is involved in regulating various biological processes such as apoptosis, cell motility, metastasis, calcium signaling, and neurodegenerative diseases (Kogias et al., 2019). Earlier research has shown that EFHD2 is essential for B cell survival as it negatively influences the NF-κB pathway that prevents apoptosis and controls the release of calcium ions from the endoplasmic reticulum. Additionally, EFHD2 serves as a pivotal regulatory factor in adaptive immune responses, contributing to associated physiological processes (Kroczek et al., 2010). However, recent studies have demonstrated that EFHD2 is significantly upregulated in the intestines of healthy mice. In contrast, in experimental colitis, the deficiency of EFHD2 results in exacerbated intestinal pathological damage (Wu et al., 2024). And EFHD2 also acts as a key regulator of immune function through cytoskeletal organization, Ca^2+^ signaling, and apoptosis modulation. The potential involvement of EFHD2 in the context of intestinal inflammation is becoming increasingly apparent. Dysregulation of the cytoskeleton mediated by EFHD2 may facilitate abnormal infiltration of immune cells, leading to subsequent tissue damage. Moreover, a defining characteristic of enteritis is the compromised function of the intestinal epithelial barrier, which is often accompanied by dysregulated apoptosis. Given EFHD2’s role in modulating apoptosis in both epithelial and immune cells, it may have a direct impact on barrier integrity and the persistence of inflammation. Despite these compelling indirect observations, the specific role and underlying molecular mechanisms of EFHD2 in enteritis remain inadequately understood. The MI can be converted into inositol triphosphate (IP_3_), which connects with the IP_3_ receptor on the membrane of the endoplasmic reticulum, thereby inducing calcium channel opening. The EFHD2, a Ca^2+^-binding adaptor protein, plays a role in calcium signal transduction (Engelke et al., 2007). However, the precise nature of the direct association between MI and EFHD2 remains to be elucidated, and further investigation is warranted to uncover the underlying mechanisms.

Grass carp, a freshwater fish with substantial economic importance, ranks as the species with the highest global production among freshwater fishes (FAO, 2024). To improve economic efficiency and boost production, grass carp farming often involves higher stocking densities and reduced feed costs, which can pose significant challenges to the maintenance of intestinal homeostasis. In inflammatory bowel disease (IBD) research, the dextran sulfate sodium (DSS)-induced model is widely considered one of the most frequently employed experimental models because of its ease of use, cost-effectiveness, and high resemblance to ulcerative colitis (Yang and Merlin, 2024). Therefore, this study aims to comprehensively assess the impact of dietary MI on the intestinal morphology of DSS-induced grass carp, as well as to investigate the underlying mechanisms by which it prevents intestinal inflammation through the regulation of programmed cell death pathways.

## 2 Materials and methods

### 2.1 Animal ethics statement

All procedures involving animals received approval from the Animal Care Advisory Committee at Sichuan Agricultural University (No. WMQ-2022114015).

### 2.2 Experimental design and DSS-induced model

The formula for the test diet, described in Table 1, was the same as in previous research (Wang et al., 2025a, b). In the six experimental diets, the MI levels were 35 (basal diet, deficient group), 98, 195, 292, 389, and 487 mg/kg diet. The grass carp weighing 704.84 ± 0.91 g were assigned at random to six different groups, with each group consisting of three replicates of 25 fish. During the trial period, four meals (07:00, 11:00, 15:00, and 19:00) were provided per day for 8 weeks, and no grass carp died. After the growth experiment, 12 fish from each group were randomly selected for the subsequent DSS-induced enteritis experiment. In this study, four concentrations of DSS (1%, 3%, 5%, and 10%) were administered via rectal injection to evaluate their effects. Injection intervals were set at 12 or 24 h, and the injection regimen was maintained for 1 to 3 d during the pre-test phase. A total of seven experimental groups were established, with the phosphate-buffered saline (PBS)-injected group serving as the control (PBS + 35 mg/kg). The remaining six groups were DSS-induced colitis models. Based on varying dietary MI concentrations, these groups were designated as DSS + 35, DSS + 98, DSS + 195, DSS + 292, DSS + 389, and DSS + 487 mg/kg, respectively. Based on the pre-experimental results (DSS concentrations of 1%, 3%, 5%, and 10%; injection intervals of 12 or 24 h; treatment duration of 1–3 d), the final experimental protocol was established as follows: 5% DSS was administered via rectal injection once every 24 h for three consecutive days. Subsequently, intestinal tissue samples were collected. Some of the samples were fixed in 4% paraformaldehyde and 2.5% glutaraldehyde for histological analysis, whereas the leftover samples were immediately frozen in liquid nitrogen and kept at −80 °C for further experimental examination.

**Table 1:** Composition and nutrient levels of the basal diet (air-dry basis, g/kg).

Ingredients	Content	Nutrients^5^	Content
Fish meal (66.25% CP)	100.00	Crude protein	267.24
Casein (75.87% CP)	170.00	Crude lipid	41.81
Gelatin (82.68% CP)	73.98	ω-3 PUFA	9.95
Fish oil	20.20	ω-6 PUFA	9.65
Soybean oil	18.20	Available P	4.00
Corn starch	188.38	Organic matter	853.50
α-Starch	300.00	Gross energy, MJ/kg	17.57
Microcrystalline cellulose	65.00		
CaH_2_PO_4_	11.30		
Mineral premix^1^	20.00		
Vitamin premix (myo-inositol free)^2^	10.00		
Choline chloride^3^	10.00		
Myo-inositol premix^4^	10.00		
Butylated hydroxyanisole	0.15		
L-Thr (97.5%)	0.91		
L-Trp (98%)	1.88		
Total	1000.00		

PUFA = polyunsaturated fatty acids.

1 Per kg of mineral premix: MnSO_4_·H_2_O (31.8% Mn), 2.66; MgSO_4_·H_2_O (15.0% Mg), 256.79; FeSO_4_·H_2_O (30.0% Fe), 12.61; ZnSO_4_·H_2_O (34.5% Zn), 8.87; CuSO_4_·5H_2_O (25.0% Cu), 0.95; Ca (IO_3_)_2_ (3.2% I), 1.56; selenium yeast (0.2% Se) 13.65. All ingredients were diluted with corn starch to 1 kg.

2 Per kg of vitamin premix (g/kg):vitamin A (500 IU), 0.44; vitamin D_3_ (500 IU), 0.19; vitamin E (50%), 25.50; vitamin K_3_ (50%), 0.38; vitamin B_12_ (1%), 0.94; D-biotin (2%), 1.05; folic acid (95%), 0.17; thiamin nitrate (98%), 0.11; vitamin C (95%), 9.77; nicotinic acid (99%), 3.44; calcium pantothenate (93.1%), 4.42; riboflavin (80%), 0.73; vitamin B_6_ (98%), 0.55. All ingredients were diluted with corn starch to 1 kg.

3 Per kg of choline chloride, which contains choline chloride (50%) 306.71 g, the rest is diluted with corn starch to 1 kg.

4 Provided the following per kg of myo-inositol (MI) premix: from treatments 1 to 6, the supplementation of MI at 0.000, 0.067, 0.171, 0.274, 0.378, 0.481 g, respectively, and filled with microcrystalline cellulose to 1 kg.

5 Available P, ω-3 PUFA, and ω-6 PUFA were calculated by NRC (2011).

The levels of crude lipid (SN-00051 B, Chengdu Shuniu Technology Co., Ltd., Chengdu, Sichuan, China) and crude protein (OLB9870A, Jinan Olabo Technology Co., Ltd., Jinan, Shandong, China) in the dried samples were analyzed using the Soxhlet extraction method (method 2003.05) and the Kjeldahl procedure (method 990.03), following the guidelines set by the AOAC (2005). The dry matter content was determined by drying the samples at 105 °C in an oven (DGT-G250, Hefei Huadeli Scientific Equipment Co., Ltd., Hefei, Anhui, China) for 4 h (method 930.15) (AOAC, 2005). Standard procedures of the method 942.05 (AOAC, 2005) were used to determine crude ash (KSY-12D-16 A, Yingshan Jianli Electric Furnace Manufacturing Co., Ltd., Yingshan, Hubei, China). The organic matter (OM) content in the feeds was determined by subtracting the crude ash content from the dry matter. Gross energy was analyzed precisely using an oxygen bomb calorimeter (Model 6400, Parr Instrument Company, Moline, IL, USA), in accordance with the ISO 9831:1998 standard protocol (ISO, 1998). Available P, ω-3 PUFA, and ω-6 PUFA were calculated by NRC (2011).

### 2.3 Histological and immunofluorescence

For histological observation, 4% paraformaldehyde-fixed intestinal tissues were taken and rinsed under running water to remove residual fixative. Tissue specimens were processed through dehydration in gradient alcohol, followed by embedding in paraffin wax. Subsequent hematoxylin-eosin (H&E) and periodic acid schiff (PAS) staining was performed. The structural characteristics of intestinal tissues were then examined under a Nikon TS100 optical microscope (Nikon Corporation, Tokyo, Japan). Immunofluorescence (IF) staining was carried out on deparaffinized sections, which were incubated with blocking buffer following antigen retrieval treatment. Subsequently, the slides were incubated with the primary antibody at 4 °C overnight. Following the primary antibody incubation, the slides were exposed to fluorescent secondary antibodies in the dark for 1 h. In the final step, anti-fluorescence quenching tablets containing 4′,6-diamidino-2-phenylindole (DAPI; G1407, Servicebio Technology Co., Ltd., Wuhan, Hubei, China) is dropped to seal the slide and cover the cover glass. A confocal microscope and an inverted fluorescence microscope to capture images and used ImageJ (ImageJ 1.53e, National Institutes of Health, Bethesda, MD, USA) to quantify the results.

### 2.4 Biochemical analysis in blood and intestines

The content of D-lactic acid (D-LA; A019-3-1, Nanjing Jiancheng Bioengineering Institute, Nanjing, Jiangsu, China) in serum samples and the activity of diamine oxidase (DAO; BC1285, Beijing Solarbio Science & Technology Co., Ltd., Beijing, China) were determined using specific detection kits. Immunoturbidimetric analysis was employed to determine the concentrations of complement proteins component 3 (C3) and component 4 (C4) in intestinal specimens (Zhejiang Yilikang Biological Technology Co. Ltd., Wenzhou, Zhejiang, China). Additionally, the activities of LZM (A050-1-1), AKP (A059-2-2), Caspase-3 (G015-1), Caspase-8 (G017-1), and Caspase-9 (G018-1) were assessed using respective kits (Nanjing Jiancheng Bioengineering Institute, Nanjing, Jiangsu, China). Determination was performed using a full-wavelength microplate reader (SpectraMax 190, Molecular Devices LLC., San Jose, CA, USA).

### 2.5 Lysosomal extraction

Endosome extraction was carried out using a kit (BB-36714, Bestbio Biotechnology Co., Ltd., Shanghai, China), according to the manufacturer’s guidelines. Briefly, 100 mg tissue sample was used, to which 1 mL cold reagent A was added. The sample was kept on ice for 10 min and then subjected to homogenization. The homogenized sample was centrifuged at 1000 × *g* for 5 min at 4 °C. The supernatant was carefully removed and collected, and the pellet was discarded. The supernatant was further processed by centrifugation at 6000 × *g* for 10 min at 4 °C. After discarding the pellet, the supernatant was subjected to an additional centrifugation step at 10,000 × *g* at 4 °C for 10 min. The resulting supernatant was retained, and the pellet was discarded. Finally, the supernatant was centrifuged at 20,000 × *g* at 4 °C for 10 min. The supernatant was removed carefully, and the remaining pellet was retained. Cold reagent B (250 μL) was then added to the pellet, and the mixture was incubated at 4 °C overnight. The next day, the sample underwent centrifugation at 10,000 × *g* for 40 min at 4 °C. The supernatant was carefully removed, and the pellet was collected for downstream experiments.

### 2.6 Quantitative real-time PCR (qRT-PCR) analysis

Total RNA was extracted using the Trizol reagent (Dalian Takara Biotechnology Co., Ltd., Dalian, Liaoning, China). The preparation of synthesized complementary DNA (cDNA) involved using a cDNA synthesis kit (Vazyme Biotech Co., Ltd., Nanjing, Jiangsu, China). The QuantStudio 5 Flex system was used to perform qRT-PCR (Applied Biosystems, Thermo Fisher Scientific Inc., Waltham, MA, USA). The final reaction mixture, with a total volume of 10 μL, was prepared using the Fast SYBR Green qPCR Master Mix kit (Aidlab Biotechnologies Co., Ltd., Beijing, China). The reaction procedure was: 95 °C, 30 s; 95 °C, 10 s; 60 °C, 30 s; 40 cycles. Using the 2^−ΔΔCt^ approach (Livak and Schmittgen, 2001), gene expression was quantified, with β-actin acting as the internal control. The full names of the abbreviations of all genes can be found in Table S1. Refer to Table S2 for the primer sequences.

### 2.7 Western blotting

The muscle samples were used for total protein extraction through radioimmunoprecipitation assay (RIPA) lysis buffer (P0013B, Shanghai Biyuntian Biotechnology Co., Ltd., Shanghai, China). After separating the protein samples using sodium dodecyl sulfate–polyacrylamide gel electrophoresis (SDS-PAGE), they were transferred to polyvinylidene fluoride (PVDF) membranes and incubated with primary antibodies overnight at 4 °C Following incubation with the primary antibody, the PVDF membrane was treated with the secondary antibody. The protein bands were detected using an enhanced chemiluminescence (ECL) reagent, and the obtained images were subsequently analyzed with ImageJ software (1.53e). The full names of the abbreviations of all protein can be found in Table S1. Details about the antibodies and dilution ratio can be found in Table S3.

### 2.8 Statistical analysis

The data was analyzed using SPSS 27.0 (SPSS Inc., Chicago, IL, USA) using one-way analyses of variance (ANOVA) and Duncan’s multiple comparisons. At the same time, the orthogonal polynomial comparison method is used to analyze whether there is a significant linear or quadratic trend. According to the statistical model:

$$Y_{ij}=\mu +T_{i}+\varepsilon_{ij}\text{,}$$

where *Y*_*ij*_ is the observation of dependent variables; *μ* is the overall mean; *T*_*i*_ is the group difference; and *ε*_*ij*_ is the residual error for the observation. The independent samples *t*-test was employed to evaluate if any differences existed between the PBS 35 mg/kg and DSS 35 mg/kg. Pearson correlation analysis was employed to evaluate the linear relationships between variables. The results are presented as mean and standard error of the mean (SEM). The statistical significance level is *P* < 0.05, and the extremely significant level is *P* < 0.001. Images were generated using Origin 2021 and GraphPad Prism 8 software.

## 3 Results

### 3.1 Intestinal development index

Table 2 displays the impact of MI on the intestinal development index of adult grass carp. With the increase in dietary MI levels, the intestinal development indices ILI (*P* = 0.044) and ISI (*P* < 0.001) initially increased and subsequently decreased, peaking at 292 and 195 mg MI/kg, respectively.

**Table 2:** Effects of diets with myo-inositol (MI) on the intestinal development of adult grass carp.

Item	Dietary MI levels, mg/kg diet						SEM	*P*-value		
	35	98	195	292	389	487		ANOVA	Linear	Quadratic
FBW, g/fish	1008.03^c^	1295.47^ab^	1318.68^a^	1331.69^a^	1330.13^a^	1218.53^b^	23.985	<0.001	0.007	<0.001
ILI^1^, %	1.62^b^	1.64^b^	1.71^ab^	1.78^a^	1.73^ab^	1.68^ab^	0.016	0.044	0.053	0.020
ISI^2^, %	1.62^b^	1.86^a^	1.79^a^	1.77^a^	1.58^b^	1.52^b^	0.028	<0.001	0.003	<0.001

FBW = final body weight; ILI = intestinal length index; ISI = intestinal somatic index; SEM = standard error of the mean.

Within a row, means without a common superscript letter differ at *P* < 0.05, *n* = 6.

1 ILI (%) = 100 × Intestinal length/Body length.

2 ISI (%) = 100 × Intestinal weight/Body weight.

### 3.2 Blood permeability and intestinal immune-related parameters

Table 3 illustrates the influence of MI on blood permeability and intestinal immune-related parameters in adult grass carp. In the DSS-induced colitis model, the activity of DAO in the MI-deficient group (DSS + 35 mg MI/kg) was significantly elevated compared to the other MI-supplemented groups (*P* < 0.001). Moreover, the content of DLA in the 292–487 mg MI/kg range was significantly lower compared to that in the deficient group (*P* < 0.001). Findings from the independent-sample *t*-test indicated that DSS treatment in the MI-deficient group had a notable impact on serum DAO activity and DLA content compared to the PBS control group (*P* < 0.05).

**Table 3:** The blood permeability and intestinal immune-related parameters of adult grass carp.

Item	PBS	DSS						SEM	*P*-value		
	Dietary MI levels, mg/kg diet								ANOVA	Linear	Quadratic
	35	35	98	195	292	389	487				
**Blood**											
DAO, U/L	228.25	302.57^a∗^	263.27^ab^	244.06^b^	219.76^c^	230.94^c^	223.05^c^	5.114	<0.001	<0.001	<0.001
DLA, μmol/L	43.38	56.42^a∗^	54.31^a^	52.97^a^	42.86^b^	45.97^b^	47.72^b^	0.961	<0.001	<0.001	0.018
**Intestine**											
LZM, U/mg prot	188.93	106.46^d∗^	122.02^c^	144.81^ab^	150.85^a^	142.59^ab^	134.12^b^	4.032	<0.001	<0.001	<0.001
AKP, U/mg prot	235.95	154.40^d∗^	165.51^cd^	173.99^c^	209.80^a^	202.83^ab^	191.28^b^	4.511	<0.001	<0.001	<0.001
C3, mg/g prot	32.53	17.45^c∗^	17.54^c^	18.80^c^	26.39^a^	25.82^a^	21.22^b^	0.841	<0.001	<0.001	<0.001
C4, mg/g prot	30.98	24.71^d∗^	25.48^cd^	27.35^b^	29.19^a^	27.80^ab^	27.19^bc^	0.376	<0.001	<0.001	<0.001
Caspase-8, U/mg prot	12.39	27.30^a∗^	24.30^b^	24.05^d^	15.73^c^	18.26^c^	19.10^c^	0.732	<0.001	<0.001	<0.001
Caspase-9, U/mg prot	13.08	28.72^a∗^	27.17^b^	21.05^c^	19.91^c^	17.77^d^	19.68^c^	0.793	<0.001	<0.001	<0.001
Caspase-3, U/mg prot	17.62	32.42^a∗^	27.82^b^	21.56^d^	20.42^d^	23.01^c^	23.49^c^	0.729	<0.001	<0.001	<0.001

The intestinal immune parameters, including LZM activity and C3 content, were significantly higher in the 195–292 mg MI/kg group in comparison to the MI-deficient group (*P* < 0.001). In addition, AKP activity and C4 content were significantly elevated in the 195–487 mg MI/kg range when compared to the deficient group (*P* < 0.001). Findings from the independent-sample *t*-test indicated that DSS treatment had a notable impact on intestinal immune parameters when compared to both the MI-deficient group and the PBS control group (*P* < 0.001).

### 3.3 Enteritis morbidity and intestinal morphology

**Fig. 1:**
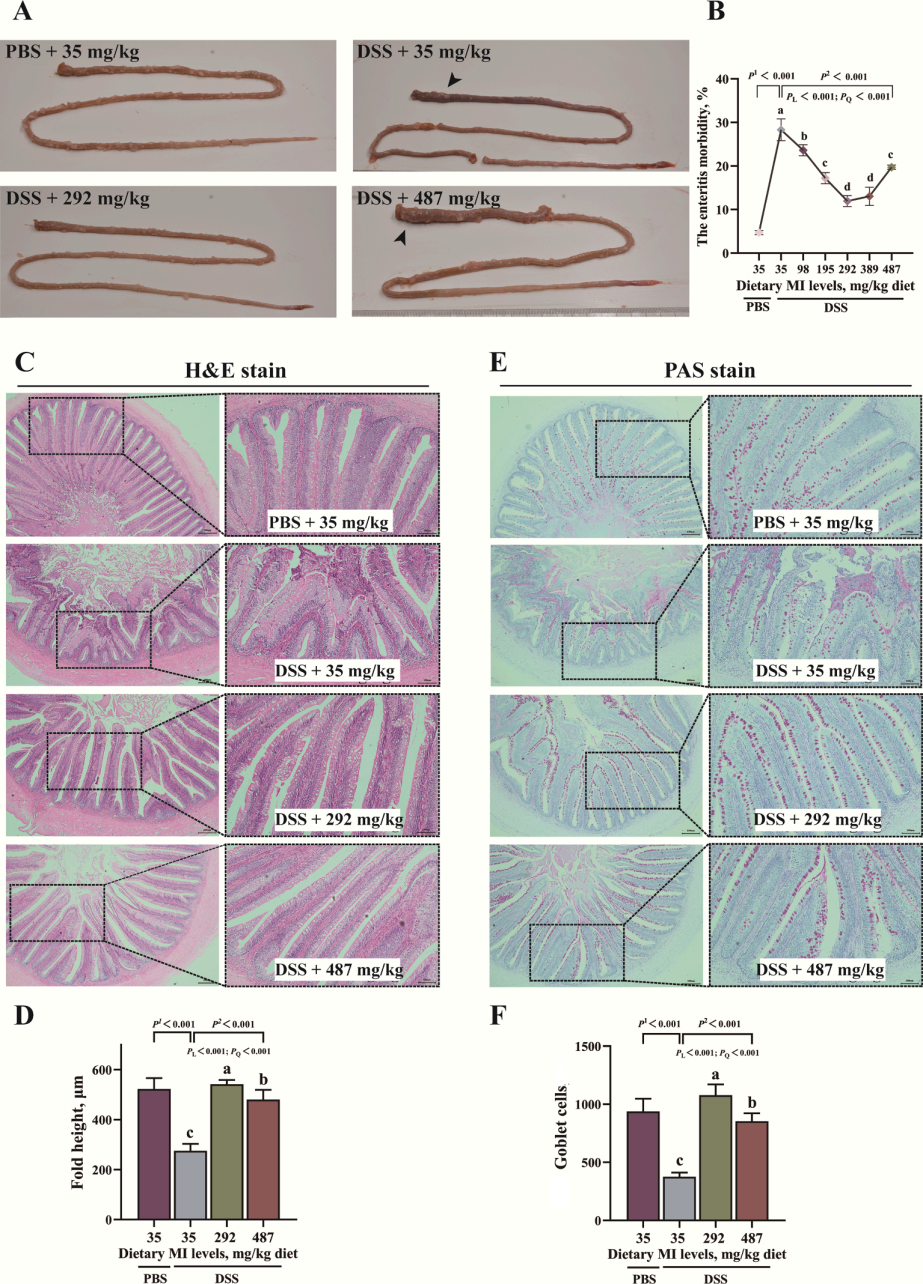
The effects of *myo*-inositol (MI) deficiency on intestinal morphology and goblet cell counts in grass carp with DSS-induced enteritis. (A) Intestinal morphology. (B) Incidence of enteritis. (C) Hematoxylin and eosin stain (H&E) staining of intestinal tissues from adult grass carp (magnification: left, 10×; right, 20×) and (D) its quantitative analysis. (E) Goblet cells alcian blue periodic acid Schiff (PAS) staining of intestinal tissues from adult grass carp (magnification: left, 10×; right, 20×) and (F) its quantitative analysis. PBS = phosphate-buffered saline; DSS = dextran sulfate sodium. *P*^1^ was the *t*-test *P*-value between PBS and DSS group. *P*^2^ was the ANOVA *P*-value among these MI groups. *P*_L_ represents *P*_linear_, *P*_Q_ represents *P*_quadratic_. Different lowercase letters above columns represent significant differences among treatments at *P* < 0.05 (*n* = 3).

In the model of colitis induced by DSS, the effects of MI on enteritis morbidity, intestinal morphology, and goblet cell numbers are presented in Fig. 1. In the MI-deficient group under inflammatory conditions, the intestinal tract exhibited redness and swelling. The MI supplementation group significantly reduced the incidence of enteritis. Histological analysis using H&E staining revealed shortened villi, mucosal surface erosion, increased inflammatory cell infiltration, and a reduction in goblet cell count. Compared with the deficient group, the MI-supplemented group exhibited a more intact intestinal structure and an increased number of goblet cells (*P* < 0.001). Findings from the independent-sample t-test indicated that DSS treatment in the MI-deficient group had significant effects on both intestinal structure and goblet cell distribution in comparison to the PBS control group (*P* < 0.001).

### 3.4 Intestinal permeability and cell death

**Fig. 2:**
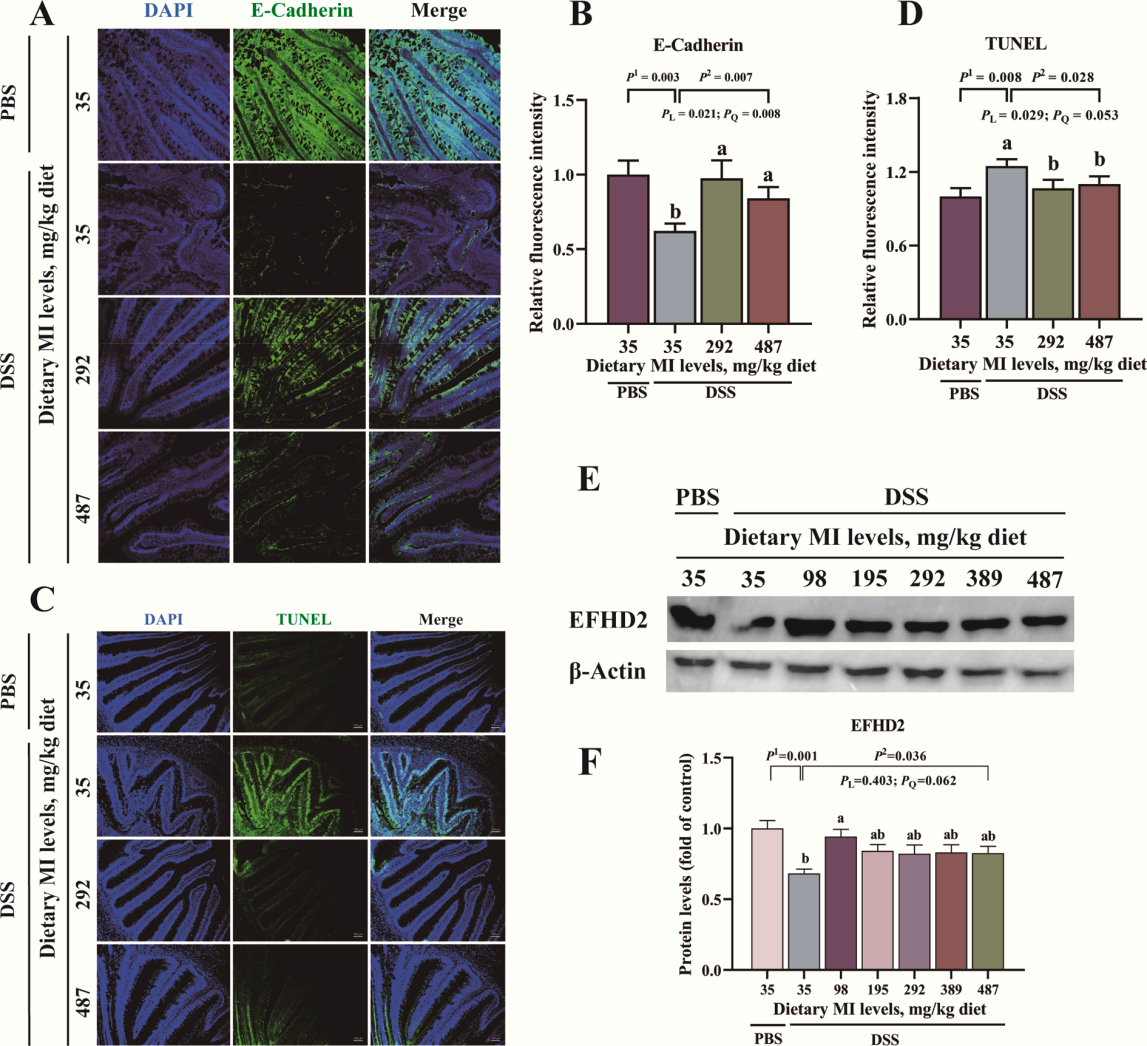
The effects of myo-inositol (MI) deficiency on intestinal permeability and cell death assessed by terminal deoxynucleotidyl transferase dUTP nick end labeling (TUNEL) staining. Immunofluorescence plots (A) and quantitative analysis (B) of intestinal E-cadherin (green) staining (20×). Immunofluorescence plots (C) and quantitative analysis (D) of intestinal TUNEL staining (10×). Intestinal EFHD2 protein expression level (E) and quantitative analysis (F). PBS = phosphate-buffered saline; DSS = dextran sulfate sodium. DAPI = 4’,6-diamidino-2-phenylindole. *P*^1^ was the *t*-test *P*-value between PBS and DSS group. *P*^2^ was the ANOVA *P*-value among these MI groups. *P*_L_ represents *P*_linear_, *P*_Q_ represents *P*_quadratic_. Different lowercase letters above columns represent significant differences among treatments at *P* < 0.05 (*n* = 6).

The effects of MI on intestinal permeability and cell death under enteritis are presented in Fig. 2. Compared with the MI-deficient group, EFHD2 protein levels were significantly elevated at 98 mg MI/kg (*P* = 0.036). E-cadherin immunofluorescence demonstrated that fluorescence intensity was reduced under MI deficiency (*P* = 0.007). In contrast, the TUNEL staining fluorescence signal was significantly enhanced in the MI-deficient group (*P* = 0.028). Findings from the independent-sample *t*-test indicated that DSS treatment in the MI-deficient group had a significant effect on both E-cadherin (*P* = 0.003) and TUNEL fluorescence intensity in comparison to the PBS control group (*P* = 0.008).

### 3.5 Necroptosis

**Fig. 3:**
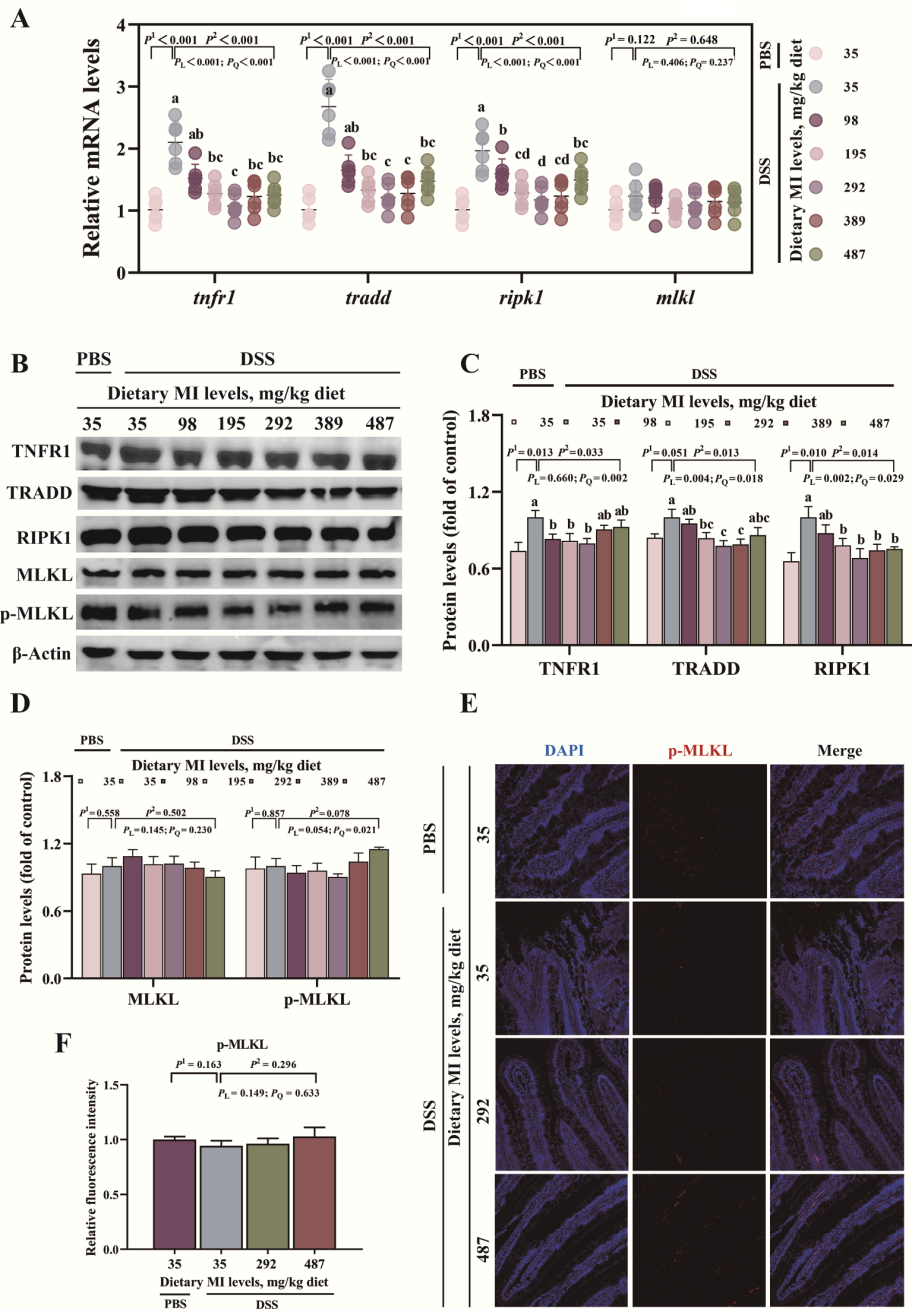
The effects of *myo*-inositol (MI) deficiency on intestinal necroptosis in DSS-induced enteritis in grass carp. (A) Expression of genes related to the apoptotic pathway. Expression (B) and quantification (C and D) of proteins related to apoptosis and programmed necrosis pathways in intestinal cells. Immunofluorescence plots (E) and quantitative analysis (F) of intestinal p-MLKL (red) staining (20×). *P*^1^ was the *t*-test *P*-value between PBS and DSS group. PBS = phosphate-buffered saline; DSS = dextran sulfate sodium; DAPI = 4’,6-diamidino-2-phenylindole. *P*^2^ was the ANOVA *P*-value among these MI groups. *P*_L_ represents *P*_linear_, *P*_Q_ represents *P*_quadratic_. Different lowercase letters above columns represent significant differences among treatments at *P* < 0.05 (*n* = 6).

The impact of MI on necroptosis under enteritis are presented in Fig. 3. In comparison to the MI-deficient group, the mRNA levels of *tnfr1*, *tradd*, and *ripk1* were significantly reduced in the MI-supplemented group (*P* < 0.001). In the 195–292 mg MI/kg group, the expression levels of TNFR1 (*P* = 0.033), TRADD (*P* = 0.013), and RIPK1 (*P* = 0.014) proteins were significantly reduced relative to the deficient group. Findings from the independent-sample *t*-test indicated that DSS treatment in the MI-deficient group had significantly influenced the expression of TNFR1, TRADD, and RIPK1 at both the protein and mRNA levels when compared to the PBS control group (*P* < 0.001). However, no notable variations were detected in the protein levels of MLKL and p-MLKL across the experimental groups (*P* = 0.078). Immunofluorescence analysis revealed no statistically significant variations in p-MLKL expression across the groups (*P* = 0.296).

### 3.6 Apoptosis

**Fig. 4:**
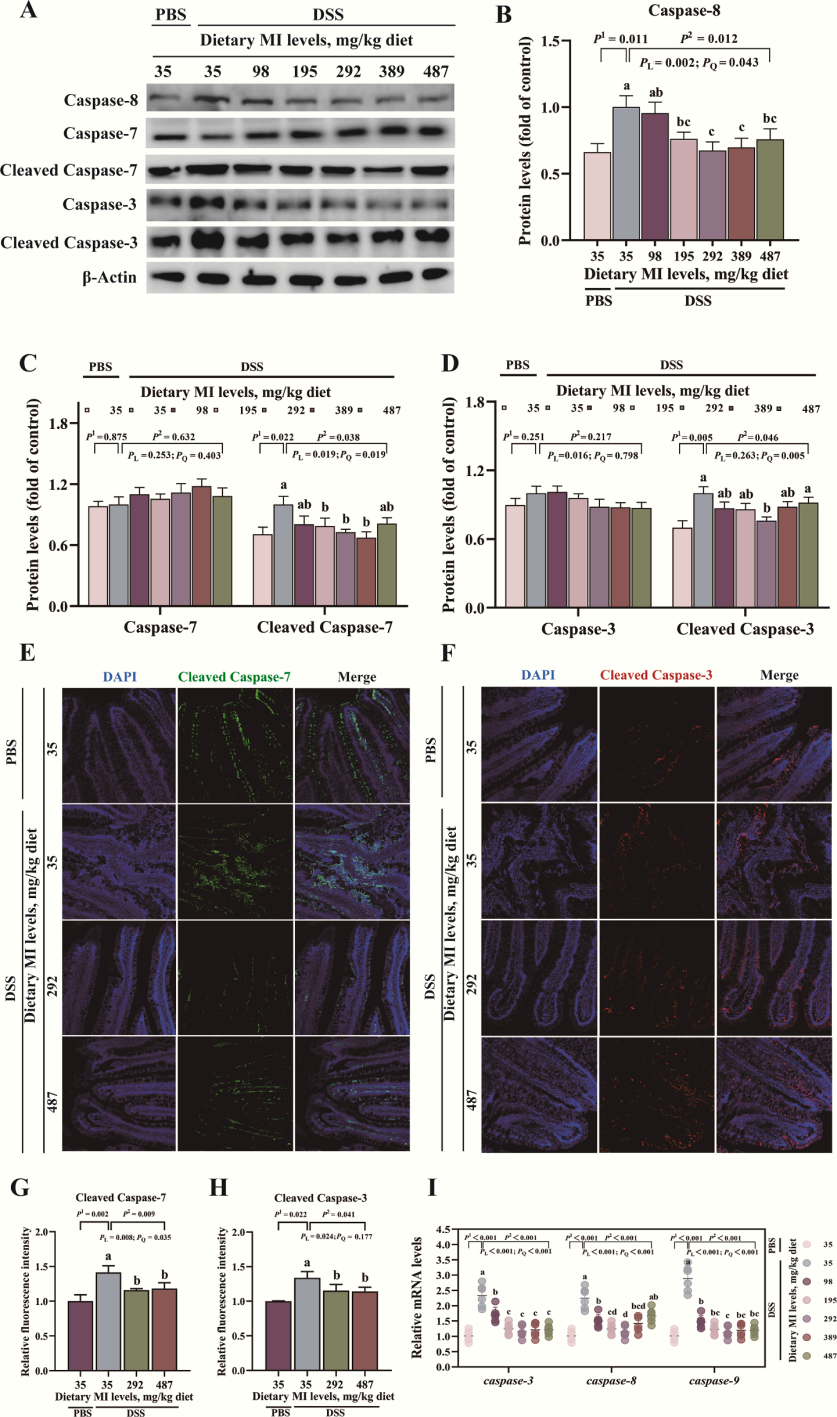
The effects of myo-inositol (MI) deficiency on intestinal apoptosis in DSS-induced enteritis in grass carp. (A) Intestinal caspase family protein expression level and quantitative analysis (B–D). Immunofluorescence plots (E) and quantitative analysis (G) of intestinal cleaved Caspase-7 (green) staining (20×). Immunofluorescence plots (F) and quantitative analysis (H) of intestinal cleaved Caspase-3 (red) staining (20×). (I) Expression levels of intestinal caspase family mRNA. *P*^1^ was the *t*-test *P*-value between the PBS and DSS groups. PBS = phosphate-buffered saline; DSS = dextran sulfate sodium; DAPI = 4’,6-diamidino-2-phenylindole. *P*^2^ was the ANOVA *P*-value among these MI groups. *P*_L_ represents *P*_linear_, *P*_Q_ represents *P*_quadratic_. Different lowercase letters above columns represent significant differences among treatments at *P* < 0.05 (*n* = 6).

In Table 3, the activities of Caspase-8, -9, and -3 in each MI supplementation group were significantly lower than those observed in the MI deficiency group under conditions of enteritis (*P* < 0.001). As shown in Fig. 4I, the relative mRNA expression levels of *caspase-8*, *-9*, and *-3* exhibited a consistent trend. Compared with the MI deficiency group, the protein levels of Caspase-8 (*P* = 0.012) and cleaved Caspase-7 (*P* = 0.038) in the 195–389 mg MI/kg group were significantly reduced. Similarly, the 292 mg MI/kg group exhibited a significantly reduced level of cleaved Caspase-3 protein compared to the deficient group (*P* = 0.046). Findings from the independent samples *t*-test indicated that DSS treatment in the MI deficiency group had significant effects on the activity of Caspase family enzymes, as well as their mRNA and protein levels, in comparison to the PBS control group.

### 3.7 TNFR1 internalization

**Fig. 5:**
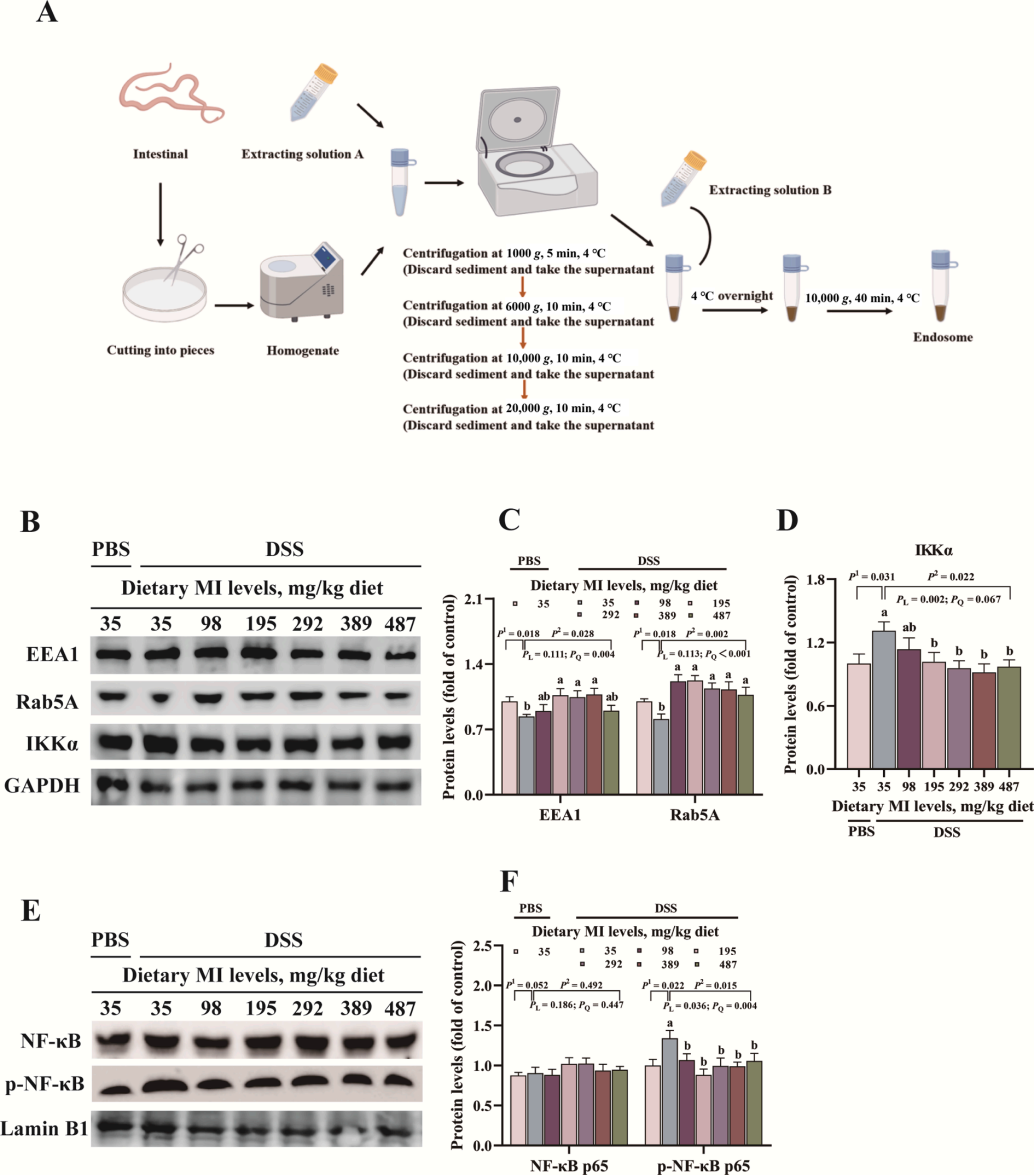
The effects of myo-inositol (MI) deficiency on intestinal endosome in DSS-induced enteritis in grass carp. (A) Intestinal endosome extraction process. (B) Endosome marker protein levels and quantitative analysis (C and D). (E) Intestinal NF-κB protein expression level and quantitative analysis (F). *P*^1^ was the *t*-test *P*-value between the PBS and DSS groups. PBS = phosphate-buffered saline; DSS = dextran sulfate sodium. *P*^2^ was the ANOVA *P*-value among these MI groups. *P*_L_ represents *P*_linear_, *P*_Q_ represents *P*_quadratic_. Different lowercase letters above columns represent significant differences among treatments at *P* < 0.05 (*n* = 6).

**Fig. 6:**
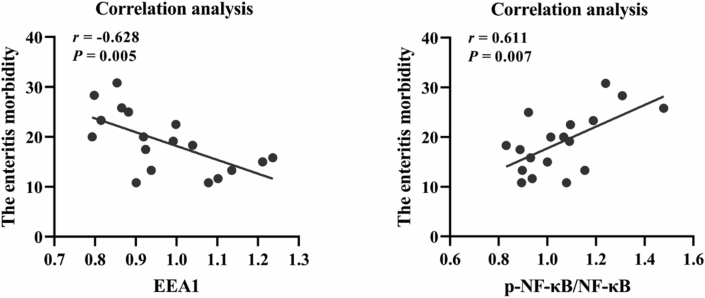
The correlation analysis of the enteritis morbidity and NF-κB pathway in grass carp.

The endosomal extraction process is illustrated in Fig. 5A. In DSS-induced colitis, the protein levels of early endosomal markers EEA1 and Rab5A were significantly increased relative to the MI-deficient group. Findings from the independent samples *t*-test indicated that DSS treatment had a significant impact on the protein levels of EEA1 and Rab5A when compared to both the MI-deficient group and the PBS control group (*P* = 0.018). In comparison to the deficiency group, the MI-supplemented group showed significantly decreased levels of IKKα (*P* = 0.022) and p-NF-κB proteins (*P* = 0.015). However, the NF-κB protein levels did not show any statistically significant differences between the groups (*P* = 0.492). Findings from the independent samples t-test indicated that DSS treatment had a notable impact on the levels of IKKα and p-NF-κB proteins when compared to both the MI-deficient group and the PBS control group. As illustrated in Fig. 6, a significant negative correlation is observed between the expression level of EEA1 and the enteritis morbidity, whereas the expression level of p-NF-κB demonstrates a significant positive correlation with the enteritis morbidity.

## 4 Discussion

### 4.1 The impact of MI on intestinal development under intestinal inflammatory conditions

In a previous study, MI deficiency disrupted MI homeostasis and protein deposition in fish. It also induced a range of physiological alterations, such as changes in myofiber size, collagen content, and lipid accumulation (Wang et al., 2025a, b). These alterations ultimately contributed to growth retardation in grass carp. At the same time, key indicators for assessing fish growth performance include body length, body width, and feeding rate (Hu et al., 2025). This study found that MI deficiency resulted in reduced body length, body width, body height, and feeding rate, thereby confirming that MI deficiency negatively affects the growth of grass carp (Wang et al., 2025a, b). Since grass carp lack a stomach, nutrient digestion and absorption rely entirely on their intestines, suggesting that intestinal development may be closely associated with growth performance (Sun et al., 2024). In this study, MI deficiency was found to reduce intestinal development indices (ILI and ISI), thus underscoring a strong correlation between MI deficiency and diminished growth performance. Furthermore, the growth and development of fish are closely associated with the health of their intestines. Therefore, the present study particularly examined the impacts of MI deficiency on intestinal health under conditions of intestinal inflammation.

The DSS-induced model is widely considered one of the most frequently employed experimental models due to its ease of use, cost-effectiveness, and high resemblance to ulcerative colitis (Yang and Merlin, 2024). Therefore, this study employed DSS as a model to simulate the protective effects of MI on intestinal health in grass carp following intestinal barrier damage caused by multiple factors in aquaculture. Additionally, the incidence of enteritis can be used as a dependable marker for assessing fish disease resistance. This study revealed that a diet deficient in MI elevated the prevalence of enteritis and exacerbated DSS-induced intestinal inflammation symptoms, such as congestion and edema. In contrast, a suitable dietary level of MI efficiently alleviated these negative impacts. Overall, these results suggest that dietary MI deficiency may induce enteritis development and undermine the structural integrity of the intestines. Moreover, the ability of fish to resist enteritis largely relies on intestinal immune function, which is closely associated with inflammatory responses. In fish, a range of immune elements, including complement proteins and naturally occurring antibacterial peptides, are essential contributors to the innate immune defense (Magnadottir, 2006). This study demonstrated that MI deficiency significantly reduced the levels of C3 and C4, as well as the enzymatic activities of LZM and AKP, suggesting that MI deficiency impairs intestinal innate immunity and disrupts immune homeostasis.

### 4.2 The impact of MI on the intestinal morphology in DSS-induced enteritis

The levels of DAO and D-LA are indicative of the severity of intestinal permeability and tissue damage (Wang et al., 2022). When intestinal injury occurs, DAO, which is produced by the intestinal mucosa, and D-LA, a metabolic product of intestinal bacteria, enter the bloodstream, leading to increased serum concentrations of these biomarkers. This study demonstrates that MI deficiency results in elevated serum levels of DAO and D-LA, indicating impaired mucosal barrier function caused by MI deficiency. Meanwhile, DAO is a highly active enzyme localized in the intestinal mucosal villi, and its activity is tightly associated with the synthesis of nucleic acids and proteins within intestinal mucosal cells (Kang et al., 2017). Histological analysis using HE staining revealed that MI deficiency leads to shortened intestinal villi, mucosal surface erosion, and increased infiltration of inflammatory cells, indicating compromised integrity of the intestinal mucosa. In a previous laboratory study, MI deficiency was demonstrated to disrupt the intestinal physical barrier function by down-regulating the expression of mRNA related to tight junctions (Li et al., 2017). Furthermore, research on pacific white shrimp (*Litopenaeus vannamei*) also reported that MI deficiency reduced both the length and width of intestinal villi, which aligns with the findings of the present study (Lu et al., 2023). Intestinal health in aquatic organisms is typically characterized by parameters such as villus height, width, and the number of goblet cells. Consequently, further investigated the impact of MI on goblet cell numbers under conditions of intestinal inflammation using PAS staining.

Goblet cells are single-cell glands within the intestinal epithelium responsible for synthesizing and secreting mucin, which establishes a protective mucosal barrier that shields epithelial cells from chemical irritants and pathogenic agents (Moniaux et al., 2001). In this study, MI deficiency was observed to reduce the number of goblet cells under conditions of intestinal inflammation. This result aligns with an earlier study, which indicated that an MI-deficient diet decreased goblet cell numbers in the intestines of hybrid grouper (♀*Epinephelus fuscoguttatus* × ♂*Epinephelus lanceolatus*), leading to disrupted intestinal morphology (Li et al., 2024). In summary, MI deficiency exacerbates DSS-induced intestinal inflammation and disrupts both intestinal morphology and the physical barrier function. E-cadherin serves an important function in tissue barrier formation and is closely associated with morphological development and homeostasis across various tissues. Therefore, further investigated the impact of MI on E-cadherin expression under conditions of intestinal inflammation.

### 4.3 The impact of MI on the transition of cell death pattern under intestinal inflammation

E-cadherin has long been recognized as a key component in maintaining intestinal epithelial homeostasis. It is predominantly localized at adherens junctions, where it plays a central role in intercellular adhesion and serves as the structural basis for the intestinal epithelial barrier (Daulagala et al., 2019). The research discovered that MI deficiency resulted in a decrease in the fluorescence intensity of E-cadherin in the intestinal epithelium of grass carp. This reduction was alleviated in the group supplemented with an appropriate amount of MI, indicating that MI deficiency exacerbates DSS-induced damage to the intestinal epithelial barrier. To explore the mechanism by which MI deficiency exacerbates intestinal epithelial damage, cell death in intestinal tissue sections was assessed via TUNEL staining. These findings suggest that MI helps maintain intestinal epithelial barrier integrity by inhibiting intestinal epithelial cell apoptosis. Apoptosis and necroptosis represent the two major forms of programmed cell death involved in intestinal inflammation. Excessive activation of either pathway can compromise the intestinal barrier, thereby promote bacterial invasion and intensifying inflammatory responses (Daulagala et al., 2019).

Recent studies have reported that EFHD2, a calcium-binding protein, is highly expressed in the intestines of normal mice but shows significantly reduced expression during DSS-induced intestinal inflammation (Wu et al., 2024). The MI is crucial in the IP_3_-calcium signaling pathway by mediating the release of intracellular calcium stores through the second messenger IP_3_, thereby influencing calcium metabolism. Based on these observations, it was hypothesized that the protective effects of MI against intestinal health in fish may be associated with EFHD2. Accordingly, further investigations were carried out. The EFHD2 is expressed across multiple cell types found in both the innate and adaptive immune systems (Dütting et al., 2011). This protein participates in key biological processes such as calcium signaling, apoptosis regulation, actin cytoskeleton organization, and synapse formation and has been implicated in the progression of various diseases (Thylur et al., 2018). In this study, during DSS-induced intestinal inflammation, the protein level of EFHD2 was found to be lower in the MI-deficient group in comparison to the PBS control group. Notably, dietary supplementation with MI restored EFHD2 expression, suggesting that the safeguarding impact of MI on intestinal inflammation may involve the upregulation of EFHD2. Furthermore, studies have indicated that the presence of two potential EF-hand domains in EFHD2 may regulate calcium-dependent activation of Caspase-9 during apoptosis (Checinska et al., 2009). However, the specific mode of intestinal cell death induced by MI during DSS-induced enteritis remains to be fully elucidated. To address this, further explored the mechanism through which MI-mediated regulation of intestinal cell death contributes to the mitigation of inflammatory enteritis.

### 4.4 The impact of MI on necroptosis and apoptosis under conditions of intestinal inflammation

As a multifunctional cytokine that promotes inflammation, TNF plays a critical role in maintaining intestinal integrity; however, it also serves as a major driver of intestinal epithelial cell death (Ruder et al., 2019). Following TNF stimulation, TRADD and RIPK1 are usually recruited by TNFR1 to form complex I on the plasma membrane, which activates NF-κB signaling. Moreover, studies have shown that serum DAO levels correlate with TNF expression, and alterations in TNF may contribute to the exacerbation of mucosal barrier injury (Zhao et al., 2014). This study demonstrates that MI deficiency causes an elevation in the mRNA and protein levels of *tnfr1*, *tradd*, and *ripk1*, suggesting that the absence of MI exacerbates intestinal epithelial cell death. To further investigate this, the necroptosis-related marker p-MLKL in intestinal tissue sections were examined. These results revealed that dietary MI supplementation does not alter the protein levels or fluorescence intensity of p-MLKL during DSS-induced enteritis, indicating that MI does not influence necroptotic cell death under inflammatory conditions. However, in previous grass carp gill cell model, MI was shown to inhibit necroptotic cell death via the MLKL signaling pathway in TNFα-induced cytotoxicity (Chen et al., 2024). This difference may be attributed to the fact that DSS-induced intestinal inflammation is accompanied by increased levels of the pro-inflammatory cytokine TNF-α. The MI may alleviate inflammatory responses through its anti-inflammatory properties and promote apoptosis to facilitate the removal of damaged cells. In gill cells, detachment from the tissue microenvironment increases their sensitivity to nutrient deficiency. As an osmotic regulator and signaling molecule, MI is integral to cellular function; its depletion directly compromises membrane stability and induces necroptotic cell death. Based on these observations, it was hypothesized that under chronic inflammatory conditions, MI may preferentially facilitate apoptosis to preserve tissue homeostasis. Consequently, further investigations into the influence of MI on apoptotic mechanisms were conducted.

Apoptosis is a Caspase-dependent process, and excessive activation of this pathway may compromise the epithelial barrier, leading to severe intestinal pathology (Edelblum et al., 2006; Günther et al., 2013). Caspase-8 and Caspase-9 are activated through distinct pathways-the extrinsic (exogenous) and intrinsic (endogenous) pathways-acting as initiator Caspases that subsequently activate effector Caspases, including Caspase-3 and Caspase-7, ultimately driving apoptotic cell death (Newton et al., 2024). In this study, during DSS-induced intestinal inflammation, the enzymatic activities of Caspase-8, -9, and -3 in the intestinal tract were elevated in the MI-deficient group in comparison to the group with PBS. In contrast, MI supplementation reduced caspase activity, suggesting that MI contributes to the mitigation of apoptotic cell death. Furthermore, the findings at the mRNA, protein, and fluorescence intensity levels related to the caspase family consistently support this conclusion. Studies have demonstrated that in mice with experimental colitis, the expression levels of both initiator (Caspase-8, -9, and -10) and effector (Caspase-3, -6, and -7) apoptotic Caspases are substantially increased relative to the control group (Yamamoto-Furusho et al., 2018), a finding that aligns with the results of the present study. Moreover, previous research revealed that MI deficiency enhances intestinal mRNA expression of *caspase-2*, *-3*, *-7*, *-8*, and *-9* following infection with *Aeromonas hydrophila* (Li et al., 2017). Collectively, these findings suggest that MI may modulate the TNFR1/TRADD/RIPK1/caspase signaling pathway to suppress apoptotic cell death.

### 4.5 The impact of MI on endosome-mediated TNFR1 internalization

Following TNF-α binding to TNFR1, the receptor complex is internalized into early endosomes via clathrin-mediated endocytosis. The endosomal microenvironment facilitates the recruitment of additional signaling molecules by TNFR1, thereby sustaining the activation of downstream pathways such as NF-κB and prolonging inflammatory signaling (Cendrowski et al., 2016). Therefore, to examine the function of MI in regulating TNFR1 endocytosis and suppressing apoptosis, further isolated endosomes. These results demonstrated that under conditions of intestinal inflammation, the protein levels of early endosome markers EEA1 and Rab5A were significantly reduced in the absence of MI. This reduction may be attributed to phosphatidylinositol 3-phosphate (PI3P), a characteristic phospholipid of early endosomes (Corvera, 2001; Stenmark and Gillooly, 2001), for which MI serves as a precursor. A deficiency in MI leads to decreased PI3P synthesis, thereby impairing the formation of early endosomes. Furthermore, impaired formation of early endosomes may prevent the effective sorting of TNFR1 following endocytosis, resulting in sustained activation of NF-κB p65 and exacerbation of intestinal inflammation. The levels of IKKα within endosomes, as well as the expression and phosphorylation of NF-κB p65 in the nucleus, confirm that endosomal signaling promotes the nuclear translocation of NF-κB. Furthermore, correlation analysis revealed a negative correlation between the expression level of EEA1 and the enteritis morbidity, whereas the expression level of p-NF-κB exhibited a positive correlation with the enteritis morbidity. In summary, MI deficiency may disrupt endosome biogenesis, thereby triggering excessive activation of the TNF/IKKα/NF-κB signaling pathway and intensifying intestinal inflammatory responses.

In summary, the findings of this study endorse the incorporation of MI (195-389 mg/kg) into the diet as a viable approach to mitigate enteritis in commercial grass carp. This strategy presents a promising solution to the challenge of enteritis in aquaculture. The potential of MI as a nutritional intervention for sustaining and enhancing intestinal health could contribute to a reduced reliance on antibiotics.

## 5 Conclusion

In conclusion, this study demonstrated that the dietary supplementation with 195–389 mg/kg of MI in fish significantly promoted intestinal development, preserved the integrity of the intestinal structure, and mitigated enteritis-induced intestinal damage. These findings elucidate the potential role of MI in maintaining and restoring intestinal barrier function and contribute to a deeper understanding of its role in supporting growth and intestinal health in grass carp.

## Credit Author Statement

**Meiqi Wang:** Writing – original draft, Visualization, Software, Investigation, Formal analysis, Data curation. **Lin Feng:** Resources, Methodology, Funding acquisition. **Pei Wu:** Methodology. **Yang Liu:** Methodology. **Hongmei Ren:** Methodology. **Xiaowan Jin:** Methodology. **Xiaoqiu Zhou:** Validation, Supervision, Resources, Methodology, Funding acquisition. **Weidan Jiang:** Writing – review & editing, Validation, Supervision, Funding acquisition, Data curation, Conceptualization.

## Declaration of competing interest

We declare that we have no financial and personal relationships with other people or organizations that can inappropriately influence our work, and there is no professional or other personal interest of any nature or kind in any product, service and/or company that could be construed as influencing the content of this paper. Xiaoqiu Zhou is an Editorial Board Member for Animal Nutrition and was not involved the editorial review or the decision to publish this article.
