# Supplementary material for: Blocking TNFR1 internalization and cell death represents a critical mechanism for alleviating enteritis in adult grass carp: The potential role of dietary myo-inositol in enhancing intestinal health
Source: Anim Nutr. 2026 Jun 2;26:716–28. doi: 10.1016/j.aninu.2025.12.020 (PMC13486875; doi:10.1016/j.aninu.2025.12.020)
Supplement: Supplementary file 1 [file mmc1.docx]

**Table S1** Abbreviations and full names of genes and proteins.

| Abbreviations | Full names |
| --- | --- |
| **Genes** |  |
| *mlkl* | mixed lineage kinase domain like pseudokinase |
| *tnfr1* | tumor necrosis factor receptor 1 |
| *tradd* | TNFR1-associated death domain |
| *ripk1* | Receptor-interacting serine/threonine-protein kinase 1 |
| **Proteins** |  |
| Caspase | Cysteine-aspartic protease |
| AKP | Alkaline phosphatase |
| EEA1 | Early endosome antigen 1 |
| EFHD2 | EF-hand domain-containing protein D2 |
| IKKα | inhibitor of nuclear factor kappa-B kinase subunit alpha |
| MLKL | mixed lineage kinase domain like pseudokinase |
| NF-κB | nuclear factor κB |
| TNF | tumor necrosis factor |
| TNFR1 | tumor necrosis factor receptor 1 |
| TRADD | TNFR1-associated death domain |
| RIPK1 | receptor-interacting serine/threonine-protein kinase 1 |
| LZM | Lysozyme |

**Table S2** The primer sequences and accession numbers of genes selected for analysis by quantitative real-time PCR.

| Genes | Forward (5′-3′) | Reverse (5′-3′) |
| --- | --- | --- |
| β-Actin | GGCTGTGCTGTCCCTGTA | GGGCATAACCCTCGTAGAT |
| *tradd* | AGTTTTCGGTGGGTTTTAAGCA | AGGCCTATCGTCTGAGAGCA |
| *tnfr1* | AGAACCGCACAGCCAGACAG | GACAGACTGGTTTGGGAAGAGC |
| *caspase-3* | GCTGTGCTTCATTTGTTTG | TCTGAGATGTTATGGCTGTC |
| *caspase-8* | ATCTGGTTGAAATCCGTGAA | TCCATCTGATGCCCATACAC |
| *caspase-9* | CTGTGGCGGAGGTGAGAA | GTGCTGGAGGACATGGGAAT |
| *ripk1* | CAGGCTCGATGCTGTAGTCA | ATCGCAGTCTCCAACCTGTG |
| *mlkl* | GGAACCGCTGTCACATGACTGTC | GTCCACCAACACTCCAGCAGAAG |

**Table S3** Primary antibodies for the western blot analyses.

| Proteins | Dilution ratio | Antibody Cat. No. | Antibody sources |
| --- | --- | --- | --- |
| EFHD2 | 1:1000 | 83264-5-RR | Hangzhou Huaan Biotechnology Co., Ltd. (Hangzhou, Zhejiang, China) |
| Cleaved caspase-3 | 1:1000 | 25128-1-AP | Hangzhou Huaan Biotechnology Co., Ltd. (Hangzhou, Zhejiang, China) |
| EEA1 | 1:1000 | A0592 | Hangzhou Huaan Biotechnology Co., Ltd. (Hangzhou, Zhejiang, China) |
| TNFR1 | 1:1000 | A1540 | ABclonal Technology Co., Ltd. (Wuhan, Hubei, China) |
| TRADD | 1:1000 | A1145 | ABclonal Technology Co., Ltd. (Wuhan, Hubei, China) |
| RIPK1 | 1:1000 | A7414 | ABclonal Technology Co., Ltd. (Wuhan, Hubei, China) |
| MLKL | 1:1000 | A5579 | ABclonal Technology Co., Ltd. (Wuhan, Hubei, China) |
| p-MLKL | 1:1000 | AP1255 | ABclonal Technology Co., Ltd. (Wuhan, Hubei, China) |
| Caspase-8 | 1:1000 | A0215 | ABclonal Technology Co., Ltd. (Wuhan, Hubei, China) |
| Caspase-7 | 1:1000 | A1524 | ABclonal Technology Co., Ltd. (Wuhan, Hubei, China) |
| Cleaved caspase-7 | 1:1000 | A23154 | ABclonal Technology Co., Ltd. (Wuhan, Hubei, China) |
| Caspase-3 | 1:1000 | A11319 | ABclonal Technology Co., Ltd. (Wuhan, Hubei, China) |
| IKKα | 1:1000 | A2062 | ABclonal Technology Co., Ltd. (Wuhan, Hubei, China) |
| p-NF-κB | 1:5000 | AP0124 | ABclonal Technology Co., Ltd. (Wuhan, Hubei, China) |
| GAPDH | 1:10000 | AC001 | ABclonal Technology Co., Ltd. (Wuhan, Hubei, China) |
| β-Actin | 1:10000 | AC050 | ABclonal Technology Co., Ltd. (Wuhan, Hubei, China) |
| Rab5A | 1:1000 | Ab124226 | Shanghai Aladdin Biochemical Technology Co., Ltd. (Shanghai, China) |
| NF-κB | 1:1000 | Ab117947 | Shanghai Aladdin Biochemical Technology Co., Ltd. (Shanghai, China) |
| Lamin B1 | 1:1500 | AF5161 | Affinity BioReagenta. (Changzhou, Jiangsu, China) |
